# A Short Manual of Surgical Operations

**Published:** 1888-03

**Authors:** 


*A Short Manual of Surgical Operations.* By ARTHUR E. J. BARKER, F.R.C.S. Pp. 423. London: LONGMANS & Co. 1887.

As well almost as could be done in the small space allotted to him, Mr. Barker has done his work. The book is a model of clear language, and of admirable arrangement. Traversing as wide a field as this book covers, very few surgeons in England could have written with an equal judgment and breadth and trustworthiness. Almost everywhere the author's work is personal; he speaks mostly of operations he has performed after having thoroughly worked them out.

Any criticism we might offer arises almost of necessity from the fact that the author has attempted a task which, in respect of the surgery of to-day, it is probably impossible for one man to perform; and certainly impossible for a dozen men to perform within the limits of a book of this size. So short are most of the descriptions of operations, that they could not be acted upon by the uneducated surgeon, while to the educated surgeon they can be little more than useful hints. No surgeon would dream of performing Oöphorectomy, or Porro's Operation, or Splenectomy, or Cholecystotomy, or Enterectomy or Gastrostomy, or indeed any one of the major operations here described, if he had to depend on the meagre accounts given in this work. Oöphorectomy is described in a little over a page, and the other operations mentioned are described in proportionate space. Surely this is scarcely enough.

As an example of subjects evidently not familiar to the author, we would select Ovariotomy. He says he has used Sir Spencer Wells's description epitomised. Now, Sir Spencer Wells's account of the operation is already a good many years old; and, as compared with the best practice of to-day, is almost antiquated. We could have dispensed with the space given to "the bull's-eye lantern" which we are advised to take among the instruments, and to the remark of how some surgeons operate through the linea semilunaris, and others through "various oblique incisions through different parts of the abdominal walls," if he had said only a few words about the cleansing of the cavity by irrigation with fluid, and had added some other equally important practical details. If we could excuse the author for stopping short at Wells for Ovariotomy, we can do nothing less than positively condemn him for again following Wells in Myomectomy and Porro's Operation. Surely the well-known surgeons who *have* done something for these operations deserve at least to have their names quoted, if not their directions. These are blemishes all the more striking when contrasted with the general excellence of the work in other departments.

In a future edition, which we hope may soon be called for, we should like to see the descriptions of all the operations expanded to an extent sufficient to meet the fullest requirements of the student and the surgeon; while we hope that those subjects with which the author is not familiar will be entrusted to other surgeons who are.

